# Supplementary material for: YUCCA-Mediated Biosynthesis of the Auxin IAA Is Required during the Somatic Embryogenic Induction Process in Coffea canephora
Source: Int J Mol Sci. 2020 Jul 3;21(13):4751. doi: 10.3390/ijms21134751 (PMC7369726; doi:10.3390/ijms21134751)
Supplement: Supplementary file 1 [file ijms-21-04751-s001.pdf]

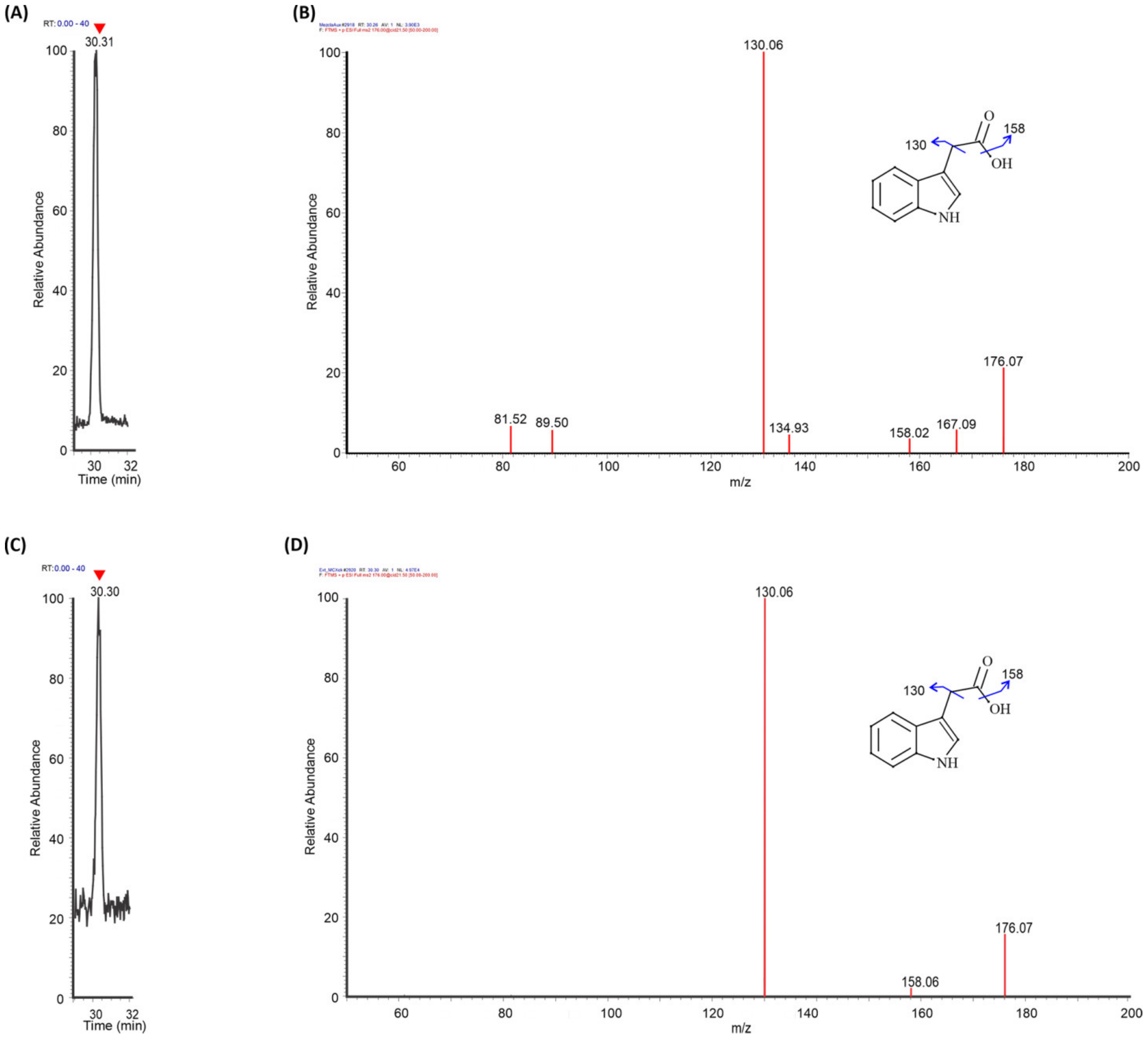

Figure S1. Chromatograms and fragmentation pattern obtained for indole-3-acetic acid by LC-MS/MS. (**A** and **B**) Chromatograms and fragmentation pattern for standard. (**C** and **D**) Chromatograms and fragmentation pattern obtained from pre-inducted leaf samples without yucasin inhibitor. Red arrowheads in chromatograms indicate the retention time for indole-3-acetic acid.

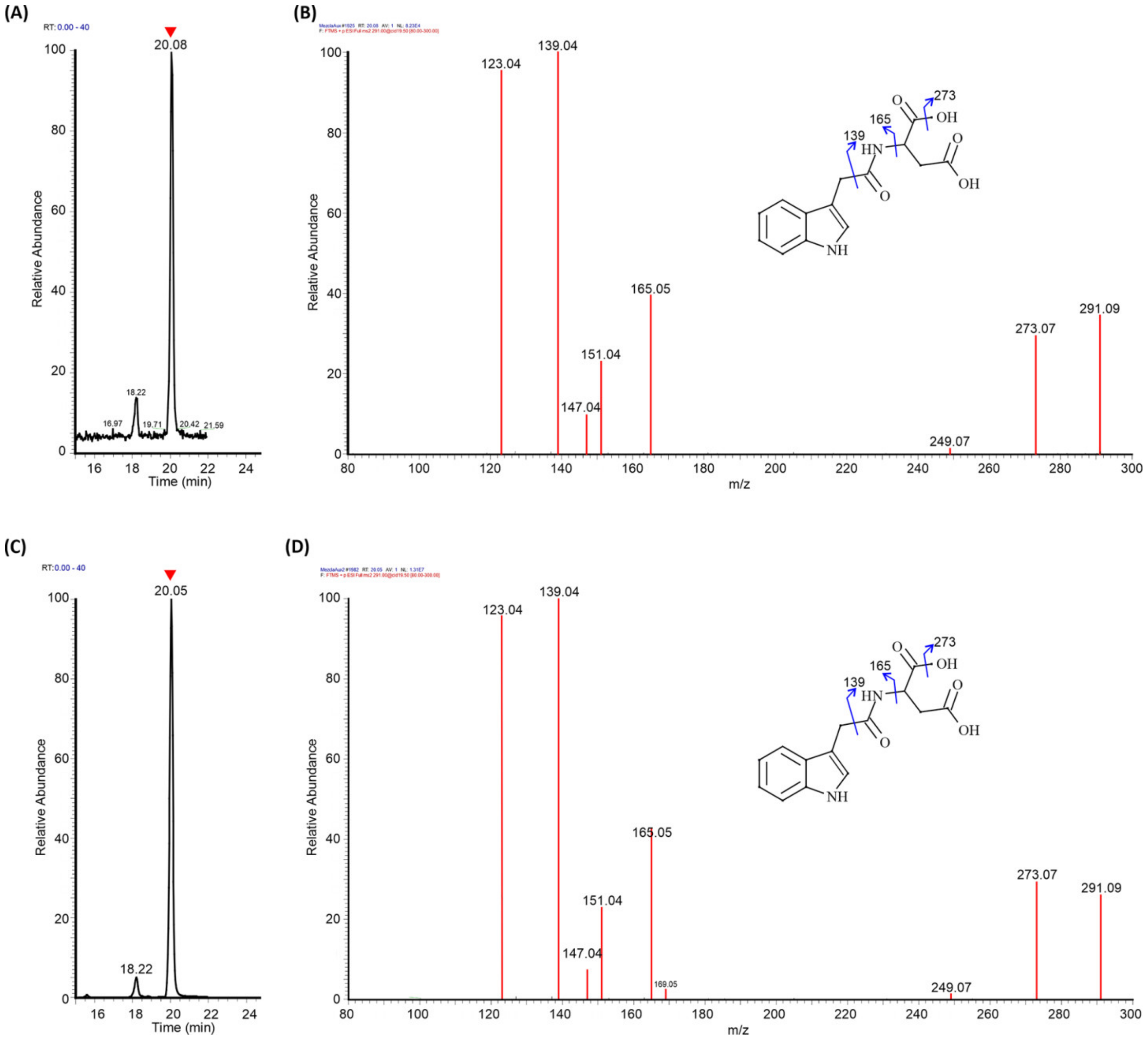

Figure S2. Chromatograms and fragmentation pattern obtained for indole-3-acetyl-L-aspartic acid by LC-MS/MS. (**A** and **B**) Chromatograms and fragmentation pattern for standard. (**C** and **D**) Chromatograms and fragmentation pattern obtained from pre-inducted leaf samples without yucasin inhibitor. Red arrowheads in chromatograms indicate the retention time for indole-3-acetyl-L-aspartic acid.

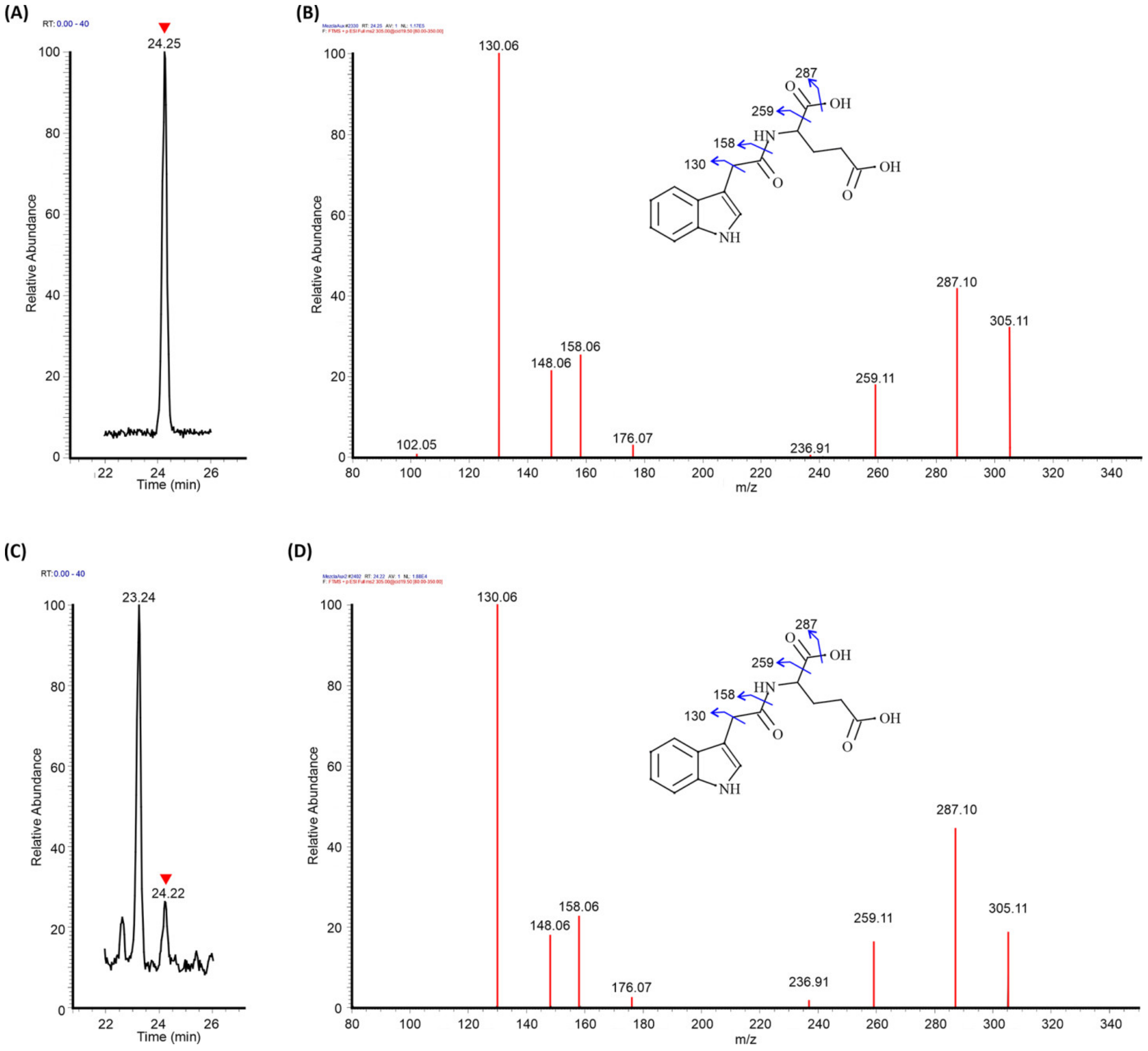

Figure S3. Chromatograms and fragmentation pattern obtained for indole-3-acetyl-L-glutamic acid by LC-MS/MS. (**A** and **B**) Chromatograms and fragmentation pattern for standard. (**C** and **D**) Chromatograms and fragmentation pattern obtained from pre-inducted leaf samples without yucasin inhibitor. Red arrowheads in chromatograms indicate the retention time for indole-3-acetyl-L-glutamic acid.

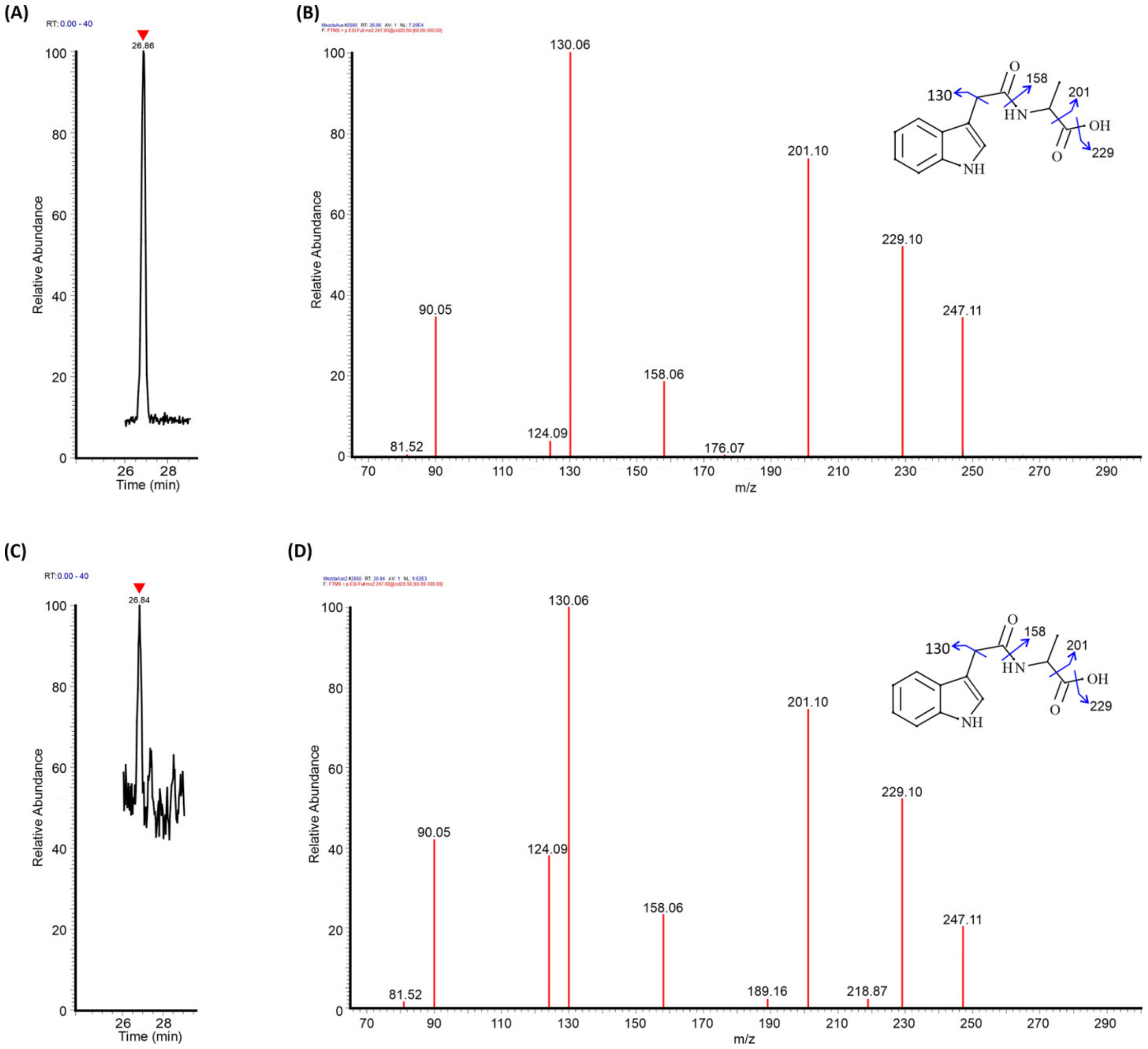

Figure S4. Chromatograms and fragmentation pattern obtained for indole-3-acetyl-L-alanine by LC-MS/MS. (**A** and **B**) Chromatograms and fragmentation pattern for standard. (**C** and **D**) Chromatograms and fragmentation pattern obtained from pre-inducted leaf samples without yucasin inhibitor. Red arrowheads in chromatograms indicate the retention time for indole-3-acetyl-L-alanine.

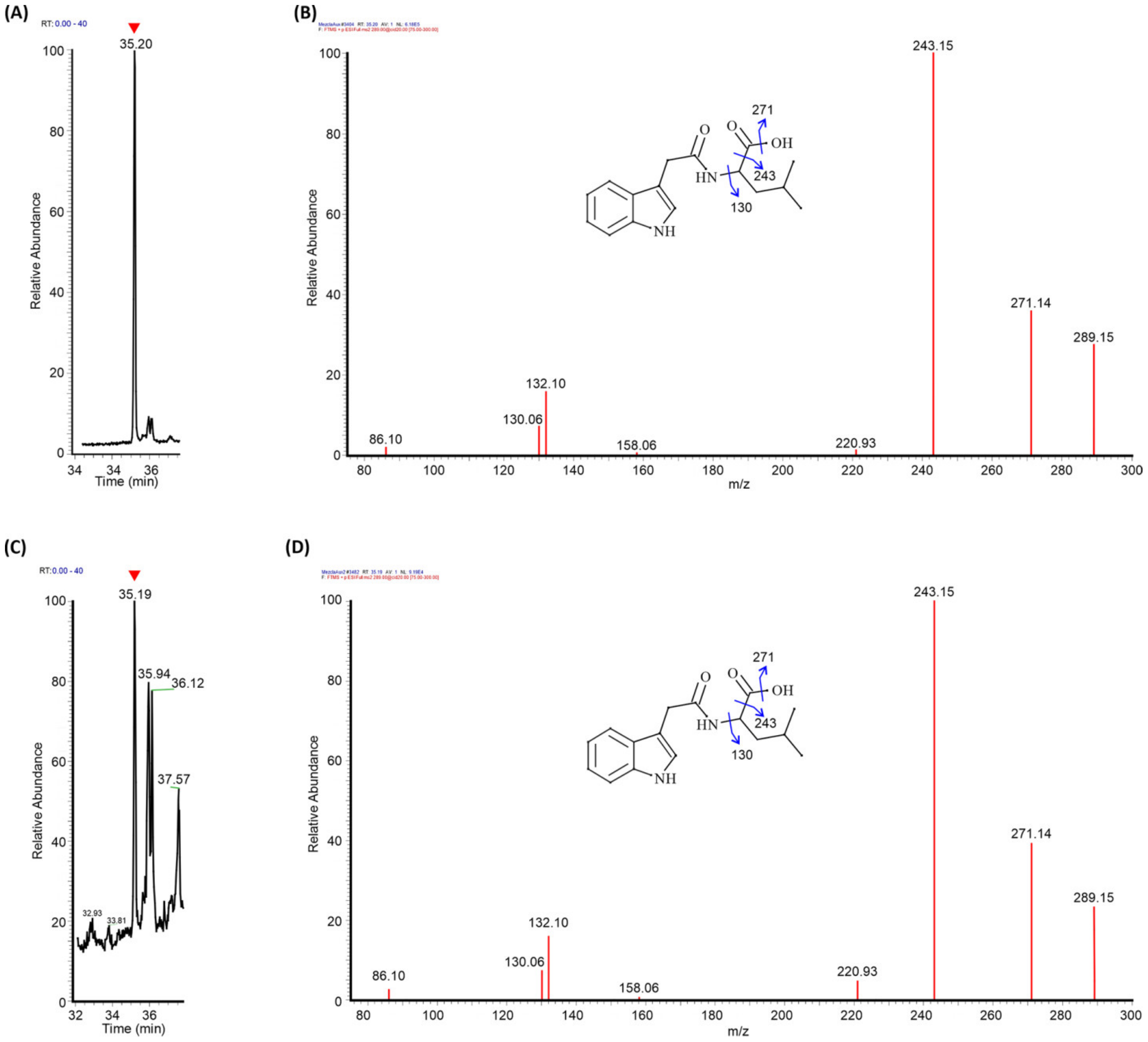

Figure S5. Chromatograms and fragmentation pattern obtained for indole-3-acetyl-L-leucine by LC-MS/MS. (**A** and **B**) Chromatograms and fragmentation pattern for standard. (**C** and **D**) Chromatograms and fragmentation pattern obtained from pre-inducted leaf samples without yucasin inhibitor. Red arrowheads in chromatograms indicate the retention time for indole-3-acetyl-L-leucine.

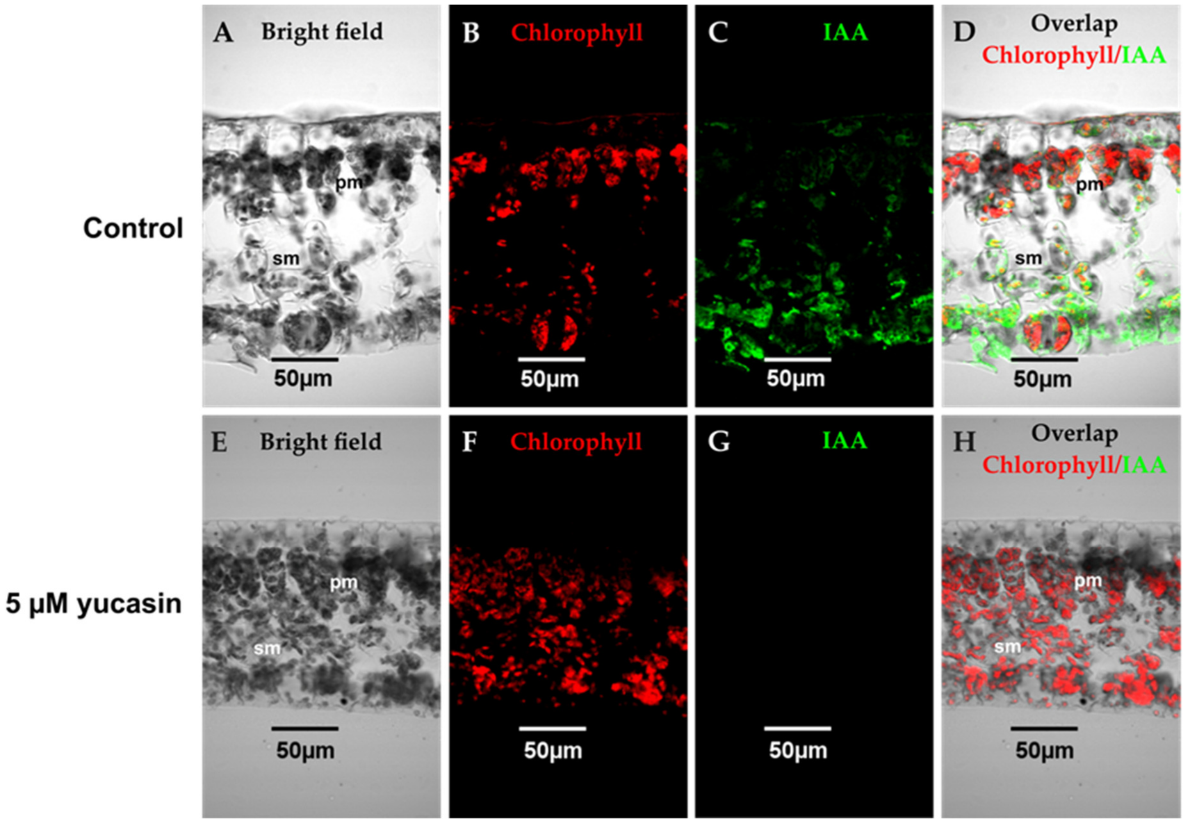

Figure S6. Immunolocalization of IAA in *C. canephora* explants treated with 5 μM yucasin. Confocal images of longitudinal sections of leaf explants of induction day (D0). Panel A and E bright field. B and F chlorophyll autofluorescence (red). C and G visualization of IAA by the Alexa 488 chromophore bonded to the antibody that recognizes the antibody-IAA (green). D and H overlapping of the chlorophyll and IAA confocal images. On the day of induction, there is not IAA signal in the explants treated with 5 μM yucasin.

Table S1. CcYUC family of genes selected for analysis by qRT-PCR and primers.

Gen	Accession	Sequence 5´-3´	Size bp
CcYUC1	Cc06_g12600	Fw: CACGGATTCTTTGGGAGGGG<br>Rv: CCACCCCAAAATGGGTAGCA	179
CcYUC1-putative	Cc06_07530	Fw: TGGTAAGGTGTTGCATTCCA<br>Rv: AGCTAGGAAGCACCCAGTGA	197
CcYUC3	Cc00_g00330	Fw: CTTTCGAGGATGGAGCTTTG<br>Rv: AAAGTGCAGGAGCAAGTCGT	165
CcYUC4	Cc11_g01360	Fw: ATTGCCTGTGGGTTGATG<br>Rv: AAGAATGACAGAAGGGACAC	101
CcYUC6	Cc08_g08920	Fw: GAGGGCTTCCCAACTTATCC<br>Rv: CTTCAATCCCACCGTCCTTA	159
CcYUC10	Cc01_g20210	Fw: TCCAAACCTAGTCCTTGAGAG<br>Rv: GACAGAACTGTTTAGCCAGG	98
CcYUC-Like	Cc00_g00340	Fw: GATCGAACTCTGACCCCTGA<br>Rv: TGGCAACTTTAGCAACATCG	184
